# Low expression of a few genes indicates good prognosis in estrogen receptor positive breast cancer

**DOI:** 10.1186/1471-2407-9-243

**Published:** 2009-07-20

**Authors:** Steven Buechler

**Affiliations:** 1 Department of Mathematics, 255 Hurley Hall, University of Notre Dame, Notre Dame, IN 46556, USA

## Abstract

**Background:**

Many breast cancer patients remain free of distant metastasis even without adjuvant chemotherapy. While standard histopathological tests fail to identify these good prognosis patients with adequate precision, analyses of gene expression patterns in primary tumors have resulted in more successful diagnostic tests. These tests use continuous measurements of the mRNA concentrations of numerous genes to determine a risk of metastasis in lymph node negative breast cancer patients with other clinical traits.

**Methods:**

A survival model is constructed from genes that are both connected with relapse and have expression patterns that define distinct subtypes, suggestive of different cellular states. This in silico study uses publicly available microarray databases generated with Affymetrix GeneChip technology. The genes in our model, as represented by array probes, have distinctive distributions in a patient cohort, consisting of a large normal component of low expression values; and a long right tail of high expression values. The cutoff between low and high expression of a probe is determined from the distribution using the theory of mixture models. The good prognosis group in our model consists of the samples in the low expression component of multiple genes.

**Results:**

Here, we define a novel test for risk of metastasis in estrogen receptor positive (ER+) breast cancer patients, using four probes that determine distinct subtypes. The good prognosis group in this test, denoted AP4-, consists of the samples with low expression of each of the four probes. Two probes target *MKI67*, antigen identified by monoclonal antibody Ki-67, one targets *CDC6*, cell division cycle 6 homolog (S. cerevisiae), and a fourth targets *SPAG5*, sperm associated antigen 5. The long-term metastasis-free survival probability for samples in AP4- is sufficiently high to render chemotherapy of questionable benefit.

**Conclusion:**

A breast cancer subtype defined by low expression of a few genes, using a minimum of statistical modeling, has significant prognostic power. This test is of potential clinical benefit in deciding a course of treatment for early stage breast cancer patients.

## Background

The decision to use adjuvant chemotherapy to treat early stage breast cancer must balance the reduced risk of recurrence with chemotherapy's toxic effects. The National Surgical Adjuvant Breast and Bowel Project trials B-14 and B-20 suggest that 85% of node-negative, ER+ patients who are treated with tamoxifen alone will be disease free for 10 years [1]. Treatment guidelines such as those from the St. Gallen consensus group [2,3] identify a small percentage of patients who can safely forgo chemotherapy; however under these guidelines, a significant number of patients undergo chemotherapy unnecessarily.

In recent years, methods of stratifying breast cancer patients according to relapse risk have been developed using multi-gene measures of mRNA concentrations. Two tests in current clinical use are the 21-gene screening panel, Oncotype DX [4,5] (Genomic Health, Redwood City, CA), and the 70-gene array-based test Mammaprint [6,7] (Agendia, Amsterdam). These tests apply to node-negative tumors with various other histopathological traits. The prospective clinical trial TAILORx [8,9] is testing the ability of Oncotype DX to identify patients who can safely forgo chemotherapy. The MINDACT trial in Europe is a similar test of Mammaprint [9,10]. Oncotype DX is used to compute the *recurrence score *[4], which is a linear combination of the expression levels of 16 genes. Patients are classified as low, intermediate or high risk using cutoffs of the recurrence score. Mammaprint is implemented with a custom microarray. The partition of patients into good and poor prognosis groups was originally accomplished by clustering with the expression values of 70 genes. Both of these tests utilize continuous measurements of mRNA concentrations of numerous genes.

The *accelerated progression relapse test*, developed here, utilizes genes that are not only connected to survival, but have expression patterns that define multiple subtypes, suggestive of distinct cellular states. Distinct expression patterns in two sets of patients suggest that different biological pathways may be active. Our approach is analogous to the familiar separation of breast cancer tumors into ER+ and ER- groups. The difference between the two groups is more than a change along a continuum; different processes are active in the two groups. Moreover, there is significant evidence that cancer in humans progresses through a series of discrete steps reflecting genetic alterations [11,12]. Genes with expression patterns that divide patients into two subtypes, one of which is enriched with poor prognosis patients, may be the most direct markers of disease progression.

**Figure 1:**
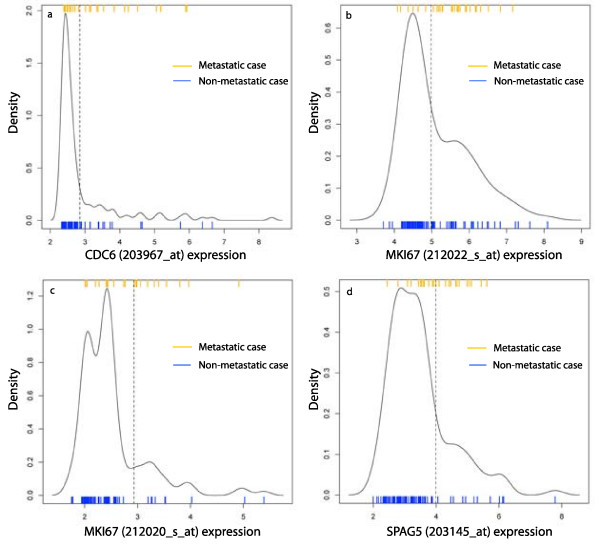
**The density distributions of the probes in AP4 divide into high and low components**. The expression values are for the four probes from the ER+ samples in the TRANSBIG cohort. The density distribution of each probe shows a large component with low baseline expression and small standard deviation, and a long right tail with elevated expression. The mixture model method applied here calculates cutoffs between the two components, indicated by the dotted vertical lines. In each case, the high component is significantly enriched with metastatic cases. The ratios of the low components are: *CDC6*, 0.95; *MKI67 *(212022_s_at), 0.85; *MKI67 *(212020_s_at), 0.89; *SPAG5*, 0.85. The ratio cutoff for being considered a multi-state probe in this cohort is 0.83.

A method akin to clustering, known as mixture models, is used to identify genes that define distinct subtypes. Unsupervised clustering is a familiar method of deriving subtypes from microarray data of cancer samples [13-15]. These applications use measures of tens or hundreds of genes. Here, we cluster samples using one gene at a time, much like the classification of samples as ER+ or ER-, ERBB2+ or ERBB2-, etc., utilizing only genes that define distinct subtypes in multiple patient cohorts. Such genes are called *multi-state *in this paper, and defined formally with mixture models in the Results section. Just as with ER status, for a multi-state probe *p *there is a threshold *c *such that the samples with expression values above *c*, denoted *p*+, form one component, and the samples with expression values below *c*, denoted *p*-, form the second component. Figure 1 plots the density distributions in one cohort of the four multi-state probes used in our prognostic test. In this paper, one component of a multi-state probe is approximately normally distributed and the other consists of a tail to the right or left.

The accelerated progression relapse test is developed using the multi-state probe methodology. The 4 probes mentioned in the abstract are multi-state, and positively correlated with relapse, across independent cohorts. The good prognosis group in the accelerated progression test, AP4-, consists of the samples in the low expression component for each of the 4 probes. The remaining samples comprise the AP4+ group. In the union of 4 independent datasets, not used to derive the subtype, the hazard ratio for distant metastasis between AP4+ and AP4- is 3.76 (95% CI 2.16 – 6.56). The 10-year metastasis-free survival probability for the AP4- group is 0.89 (95% CI 0.85 – 0.93), making systemic chemotherapy of questionable benefit.

## Methods

### Patient cohorts and data analysis

The microarray datasets used here were obtained from the Gene Expression Omnibus http://www.ncbi.nlm.nih.gov/geo/, specifically, GSE4922 (UPPS), GSE6532 (OXFD, GUYT), GSE7390 (TRANSBIG), GSE9195 (GUYT2), and GSE11121 (MZ). The codes used for the cohorts in this paper are given in parentheses. Two independent cohorts were obtained from GSE6532, for a total of 6 cohorts, with 813 ER+ samples. The OXFD cohort is the combination of OXFT and OFXU from GSE6532. (GSE6532 contains additional cohorts, coded KIT and KIU, however these cohorts were excluded since many of the patients in these groups are also found in GSE4922.) None of the patients received adjuvant chemotherapy. A summary of the clinical traits of the patients is found in Table 1. Complete descriptions can be found in the references at the Gene Expression Omnibus. Only the estrogen receptor positive tumors were used in this study. Estrogen receptor status was determined here from the microarray data, since the clinical status was assessed by different methods across the cohorts. A "+" after the code for a cohort denotes the set of ER+ samples. In all cohorts, the survival endpoint used was distant metastasis, except in GSE4922, in which it was local recurrence or metastasis. Data on metastasis for most of the samples in this cohort are found in GSE6532. All survival data was censored to 10 years so as not to distort the data due to different study lengths. In each cohort, the mRNA was extracted from primary tumors and hybridized to an array from the Affymetrix GeneChip platform hgu133a or hgu133plus2.

**Table 1:** Summary of the patient cohorts used in this study

	Uppsala	Transbig	Guys 1	Oxford	Guys 2	Mainz
Code	UPPS	TRANSBIG	GUYT	OXFD	GUYT2	MZ

GEO Series	GSE4922	GSE7390	GSE6532	GSE6532	GSE9195	GSE11121

array	hgu133a	hgu133a	hgu133plus2	hgu133a	hgu133plus2	hgu133a

# samples	249	198	87	178	77	200

# ER+	200	138	85	144	77	169
LN+/-/? on ER+	62/132/6	0/138/0	56/29/0	36/102/6	36/41/0	0/169/0

Tamoxifen	?	?	85	105	77	?

Grade 1/2/3/? on ER+	64/110/26/0	29/72/36/1	17/37/15/16	25/63/22/34	14/20/24/19	29/123/17/0

< 2 cm/≥ 2 cm on ER+	95/105	54/84	30/55	56/88	28/49	88/81

LN+/-/? = lymph node status; positive, negative, missing

Tamoxifen = number of patients receiving tamoxifen

< 2 cm/≥ 2 cm = the diameter of the tumor

The language *R *http://www.r-project.org/ was used for all statistical analyses. Survival models were fit with the *R *package *survival*. The proportional hazard condition was verified with the cox.zph function. All *p*-values in survival models refer to the *p*-value of the logrank score of a Cox proportional hazard model (CPH). A CPH is considered statistically significant if the *p*-value of the logrank score is < 0.05.

### Mixture models

Given a numeric vector, the statistical method of finite mixture models partitions the vector into components, each of which is modeled by a different density distribution. The mixture models used in this paper fit a pair of gaussian distributions to a vector. Such a model is described by a partition of the vector into components *C*_1_, *C*_2_, and a pair of gaussian distributions *g*_1_, *g*_2 _modeling the distributions of *C*_1_, *C*_2_, respectively. The modeling process simultaneously partitions the vector and selects the means, *μ*_1_, *μ *_2 _and standard deviations *σ*_1_, *σ*_2 _of the two gaussian distributions, with the goal of giving the best possible fit over all alternatives. The fitting algorithm actually produces, for each point and component, a posterior probability that the point is in that component. The point is assigned to the component whose associated posterior probability is maximal.

For a point *p *that is well-classified in, say, component 1, the posterior probability that *p *is in *C*_2 _will be very small. For convenience, posterior probabilities below a threshold *δ *are reported as 0. Following [16], we use *δ *= 10^-4^. Points that are on the boundary between the two components will have posterior probability > *δ *for both components. The "isolatedness" of, e.g., component 1 is assessed by the *ratio*, *r*_1 _= *n*_1_/*m*_1_, where *n*_1 _is the size of *C*_1 _and *m*_1 _is the number of elements with posterior probability of belonging to *C*_1 _greater than *δ*. Ratios are ≤ 1, with numbers close to 1 representing well-isolated components. Ratios will be used in this paper to measure the ability of a mixture model fit to describe distinct states.

In most instances, the components defined by a fit of a pair of gaussian distributions consist of a pair of unbroken intervals. That is, there is a cutoff *c *so that one component consists of the values <*c *and the other component the values ≥ *c*. In this way, mixture models can be used to calculate a threshold for dividing a vector into high and low components.

A standard measure of the quality of a mixture model fit is the likelihood, which is the product, over all points, of the maximal posterior probabilities. The likelihood can be used to decide, for example, if a fit with a pair of gaussian distributions is better than a fit with a single gaussian, or if a fit with Gamma distributions is better than a fit with gaussian distributions. Even better measures are AIC and BIC which adjust likelihood by the degrees of freedom. These measures play a part in defining the notion of a multi-state probe.

In this paper, mixture models were fit using the *flexmix *[16]*R *package.

## Results

The results in the paper consist of both the development of the multi-state probe methodology for survival models, and the application of this method to breast cancer.

### Multi-state probe methodology for survival models

#### GCRMA is used to calculate expression values

Expression values are computed from the CEL files with gcrma [17]. Many of the genes central to proliferation are unexpressed or expressed at a low baseline level in normal tissue. Given the prominent role played by proliferation in breast cancer progression, it is important to measure low concentration mRNA levels as precisely as possible. It was shown in [18] with spike-in data that gcrma has superior accuracy and precision to other methods in measuring low concentration mRNA. The effect on the AP4 model of using MAS5 instead of gcrma is described in the Discussion section.

Note that gcrma is applied separately to each of the 6 microarray datasets. Expression values in different datasets are never compared to each other. This allows us to include in the study datasets based on both hgu133a and hgu133plus2. A binary variable for each probe in the AP4 model is calculated as a step in forming the AP4 partition in a dataset. Whether a sample has a value 0 or 1 is based only on the probe's expression values within the dataset. In studying properties of the AP4 model we do merge the datasets of binary variables. This allows us to reference, e.g., one large validation dataset that is the union of four cohorts.

### Multi-state probes are defined with mixture models

As motivated in the Background section, distinct gene expression patterns are used here to model distinct biological states. At a basic level, mixture models can be fit to expression vectors to identify these different states. However, the natural variation in expression patterns makes it a challenge to decide which fits to multiple distributions represent distinct states and which are simply anomalies in the data. The fact that most microarray databases contain fewer than 200 samples accentuates the problem. In a preliminary study we found that, ranging over a large set of probes in one microarray database, for all but a few probes, a fit with a pair of gaussian distributions has higher likelihood than a fit with a single distribution (either gaussian or Gamma). A more stringent measure than likelihood is needed to separate those patterns that represent distinct states from noise. The phenotype we are trying to model will guide the definition of a multi-state probe.

Let *x *denote the expression vector of a gene such that increased expression is positively correlated with relapse in a cohort of ER+ breast cancer patients. Suppose that a fit to a pair of gaussian distributions produces two components, consisting of the values above a threshold *c *and the values below *c*. The high component will be enriched with metastatic cases. For a gene that significantly influences metastasis, many of the samples in the high component will be metastatic. In a representative cohort only about 25% of the patients eventually metastasize, so the high component is likely much smaller than the low good prognosis component. Instead of appearing as a pair of components of equal size, it is modeled by a large normal component and a right tail of elevated values. The degree of separation of the tail from the low component is a measure of the quality of this fit. Referring to the parameters described above, this suggests that a high value for the ratio of the low component indicates a gene with distinct states. For a gene *y *that is negatively correlated with relapse, the high component is the good prognosis group and the low component is enriched with metastatic cases. In analogy with *x*, the ratio of the high component of *y *measures the quality of this fit. In either case, the ratio of the good prognosis component is the important parameter. This is the motivation behind the following definition. Also see Figure 1.

Given a microarray database *S*, let **Y **be a large set of probes that are correlated with relapse. For each probe *p *in **Y**, fit a pair of gaussian distributions to the expression vector for *p *in *S*, and let *r*_*p *_be the ratio of the good prognosis component. Let *r*_0 _be the median of *r*_*p*_, for *p *in **Y**. A probe *p *in **Y **is *multi-state in S *if *r*_*p *_> *r*_0_.

The density distributions of the four probes in AP4 in the TRANSBIG+ cohort are plotted in Figure 1, along with the cutoffs between high and low components and indicators for metastatic cases.

An adjustment to the mixture model process is required for a probe whose distribution can be modeled with a pair of gaussian distributions in multiple ways, or when the components are broken intervals. This occurs when, as in Figure 1(c), the optimal fit is with 3 gaussian components instead of 2. However, routinely fitting expression vectors with more than 2 gaussian distributions risks over fitting the data. It is rare for the 3 component fit to be optimal across multiple cohorts. Instead, for a vector positively correlated with relapse we remove from the vector the lowest 10% of values prior to fitting with a pair of gaussian distributions. For a gene negatively correlated with relapse we trim the highest 10% of values. This correction is necessary for fewer than 5% of the probes in this study and does not effect the cutoff between components for other probes.

The parameters for the pair of gaussian distributions can be used to illustrate the quality of the fit for multi-state probes in specific datasets. Let **Y **be the 100 most significant probes in the UPPS+ cohort, as described below in the derivation of AP4. The median ratio of good prognosis components for this set is 0.89. Let *x *be the expression vector of a multi-state probe positively correlated with relapse, *g*_*L*_, *g*_*H *_the gaussian distributions of the low and high components, *μ*_*L*_, *μ*_*H *_and *σ*_*L*_, *σ*_*H *_the means and standard deviations of *g*_*L*_, *g*_*H*_, respectively, and *c *the cutoff between the low and high components. We find empirically, that for any such *x *that is multi-state, *μ*_*H *_- *μ*_*L *_> 5*σ*_*L *_and *c *- *μ*_*L *_> 2.8*σ*_*L*_. That is, all elements of the high component are above the 0.997 quantile of *g*_*L*_. This shows a high degree of separation between the components.

It is worth noting that, in some instances, a fit with a pair of Gamma distributions has a higher likelihood than a fit with gaussian distributions. However, checking the multi-state probes in one cohort in the study, the components defined by Gamma distributions and gaussian distributions are exactly the same for half of the probes and never differ by more than 2%. Thus, we choose to work only with the simpler gaussian distributions.

#### The mixture model methodology recognizes subtypes

**Figure 2:**
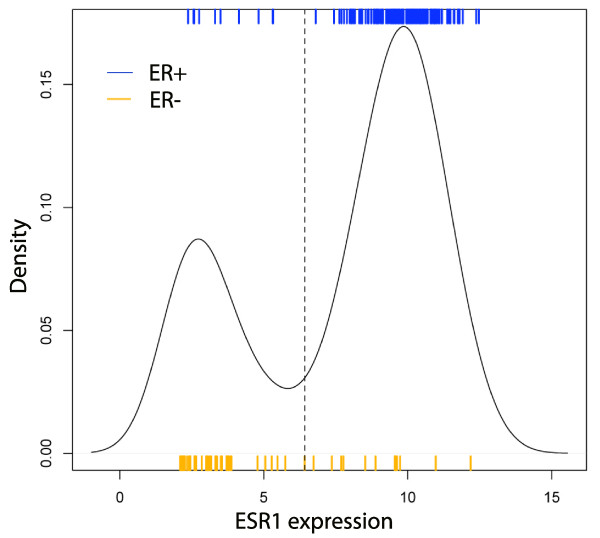
**Density distribution of ESR1 compared to ER status**. Mixture models applied to the *ESR1 *gene accurately model the clinically assessed ER status. The dotted line indicates the cutoff between high and low components as found by fitting the density distribution with a pair of gaussian distributions. The accuracy of the approximation of ER status by the high and low components is 0.88 (95% CI 0.85 – 0.92), as determined by Monte Carlo cross-validation.

To test the effectiveness of the multi-state methodology to define subtypes, it is applied to the estrogen receptor 1 gene, *ESR1*, to model ER status. In [19] it was shown that a partition of samples based on expression values of the probe *p *= 205225_at yields about 90% agreement with ER status, as assessed by immunohistochemistry (IHC). In [19], the threshold between high and low expression of the probe was determined by a fit to ER status in a training set. Here, the above mixture model methodology was applied to the expression vector of the probe *p *in the TRANSBIG cohort to find *p*- and *p*+ components. Agreement with the clinically assessed ER status was compared. This process was repeated with 1, 000 subsets of TRANSBIG, each consisting of 2*/*3 of the samples. The accuracy of the mixture model approximation was 0.88 (95% CI 0.85–0.92). At the same time, possible cutoffs for *p *were surveyed and the maximum accuracy was recorded for each of the 1, 000 subsets. The maximum accuracy of a *p *expression cutoff to reproduce ER status was 0.89 (95% CI 0.86 – 0.92). Similar results were obtained in OXFD. See Figure 2.

Thus, the mixture model approach to dividing samples into low and high components of *ESR1 *expression, based solely on the distribution of expression values, gives nearly optimal agreement with ER status as assessed by IHC.

#### Survival model development focuses on good prognosis patients

Reflecting the position that the +/- status of a multi-state probe is as informative as the raw expression values, multi-state probes will be represented in survival models as binary variables: 0 for the good prognosis component, and 1 for the other. For *X *a multi-state probe or corresponding binary variable, *gd*(*X*) denotes the good prognosis group of samples. For a probe positively correlated with relapse, *gd*(*X*) is the low expression component.

Survival models using multiple multi-state probes are defined to focus on the good prognosis samples. The partition of samples generated by a set of multi-state probes *X*_1_,...,*X*_*n *_distinguishes the samples in *gd*(*X*_*i*_), for all *i*, from the rest. Identifying the *X*_*i *_with binary variables, this partition is defined by the binary variable *Y *that is 0 when every *X*_*i *_is 0, and 1 otherwise. *Y *is denoted *X*_1 _*⋯* *X*_*n*_. The survival model generated by *X*_1_,...,*X*_*n *_is a model whose sole variable is the binary variable *X*_1 _*⋯* *X*_*n*_. Under this approach, a sample that is in the high-risk component for any variable is high risk overall. Gradations of risk in the poor prognosis group that may be defined by multiple probes are not part of this study. For *Y *the * product of multi-state probes, *gd*(*Y*) is the set of samples on which *Y *is 0.

Given a set **P **of multi-state probes and a sample cohort *S*, an optimal survival model derived from **P **is defined with a binary variable *Y *such that (1) *Y *= *X*_1 _*⋯* *X*_*n*_, for some *X*_1_,...,*X*_*n *_in **P**, (2) on *gd*(*Y*) no CPH using a single *Z *in **P **is significant, and (3) no variable *Y' *that is the * product of a proper subset of *X*_1_,...,*X*_*n *_satisfies (2). Less formally, *gd*(*Y*) is the largest set that is the intersection of good prognosis sets defined with elements of **P**, and which cannot be significantly improved by intersecting with a further element of **P**.

While multi-state probes are represented by binary variables, other probes can be tested for significance as continuous expression vectors. This is a routine step in finding an optimal model by this method. In this way, no information is lost by first considering some probes as binary variables.

### The AP4 test for metastasis in ER+ breast cancer

#### Derivation of the AP4 model

The AP4 model is derived with the ER+ samples in two cohorts as training sets, GSE4922 (denoted UPPS+) and GSE7390 (TRANSBIG+). An initial set of 100 significant probes is identified as follows: Working in UPPS+, 100 training sets are selected, each containing 2*/*3 of the samples that relapse and 2*/*3 of the samples that do not relapse. For each training set and each probe *p*, a CPH is computed using as its sole variable the expression vector of the probe restricted to the training set. For each training set, the 100 most significant probes, as measured by the logrank score *p*-values, are selected. Finally, let **Y **be the 100 probes that occur most frequently in the top 100 probes for these training sets.

A set of probes to serve as candidates for inclusion in the model is selected from **Y **as follows: Let **Y**_**up **_be the probes in **Y **that are positively correlated with relapse. Let **P**_**up **_be the probes *p *in **Y**_**up **_such that (1) *p *is multi-state in both UPPS+ and TRANSBIG+, and (2) the binary variable representing *p *is significant in a CPH in UPPS+ and TRANSBIG+. A set **P**_**dn **_of probes negatively correlated with relapse is derived correspondingly. The set of candidate probes **P **is the union of **P**_**up **_and **P**_**dn**_. Executing this procedure yields a set of 16 probes.

An optimal survival model derived from **P **in UPPS+ is generated by *CDT1 *(209832_s_at), *SPAG5 *(203145_at), *CDC6 *(203967_at), and *SNRPA1 *(216977_x_at). In TRANSBIG+ an optimal survival model derived from **P **is generated by *MKI67 *(212020_s_at), *SPAG5 *(203145_at), *PLK1 *(202240_at), *SNRPA1 *(216977_x_at), and *MKI67 *(212022_s_at). In both cases the probes are all positively correlated with relapse, hence the good prognosis group for any probe is the low expression component. As the initial model, called AP7, we choose the one generated by the 7 probes obtained from either cohort. This ensures that samples in AP7- in both UPPS+ and TRANSBIG+ have good prognosis. While the process identifies AP7, models generated by fewer of these probes perform as well in the 6 cohorts in this study. One of these tests is AP4, using 212020_s_at (*MKI67*), 212022_s_at (*MKI67*), 203967_at (*CDC6*), and 203145_at (*SPAG5*). For the cohorts in this study, refining AP4- to the smaller AP7- removes very few metastatic cases.

No continuous expression vector of a probe in **Y **is significant in a CPH on the subset AP4- in both UPPS+ and TRANSBIG+. That is, no continuous expression vector improves on the model defined discretely using the multi-state probes. Similarly, no multi-state probe negatively correlated with relapse improves this model.

#### Definition of the accelerated progression subtype in an arbitrary cohort

Given a microarray dataset for ER+ breast cancer tumors *S*, based on the Affymetrix platform hgu133a or hgu133plus2, the accelerated progression subtype is defined as follows:

1. For each of the four probes 212020_s_at (*MKI67*), 212022_s_at (*MKI67*), 203967_at (*CDC6*), and 203145_at (*SPAG5*), apply the multi-state probe methodology to divide the samples into high and low components.

2. Define AP4- to be the samples in the low component for each of the four probes; a sample is AP4+ if it is in the high component for any of the probes.

The terminology reflects the viewpoint that samples in AP4+ have elevated expression of genes that accelerate cell cycle progression; AP4- samples have baseline expression of these genes.

#### AP4 is a significant predictor of metastasis in independent cohorts

**Figure 3:**
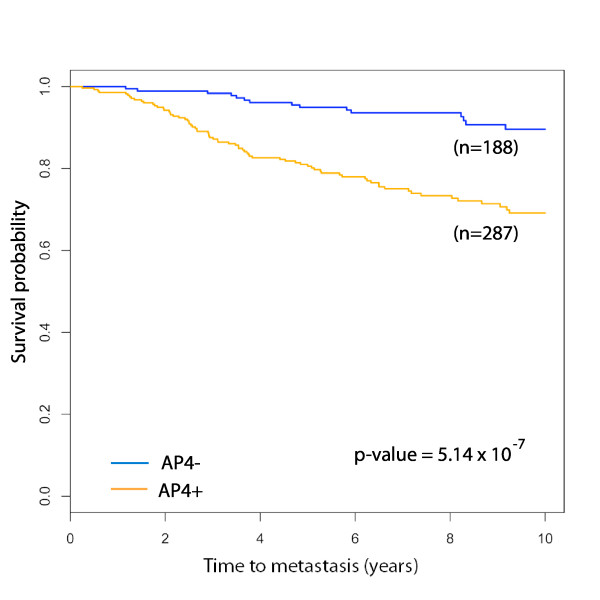
**Kaplan-Meier survival plot for AP4 in the validation set**. The validation set consists of the ER+ samples in the cohorts OXFD, GUYT, MZ and GUYT2, totalling 475 patients. The 5 and 10-year metastasis-free survival probabilities for the AP4- group are 0.95 (95% CI 0.92 – 0.98) and 0.89 (95% CI 0.85 – 0.93), respectively. The corresponding probabilities for the AP4+ group are 0.77 (95% CI 0.73 – 0.81) and 0.65 (95% CI 0.60 – 0.70). The hazard ratio between AP4+ and AP4- is 3.76 (95% CI 2.16 – 6.56).

The AP4 model is validated in the four independent cohorts, OXFD+, GUYT+, GUYT2+ and MZ+, none of which is used to derive the model. The performance statistics for the model in each cohort are reported in Table 2. The mean 10-year metastasis-free survival probabilities of the AP4- groups is 0.92, and AP4 is statistically significant in three of the four cohorts. The binary variables defining AP4 in each cohort are merged to form a single variable ranging over the union of the four cohorts. A Kaplan-Meier plot for AP4 in the union of these cohorts is found in Figure 3.

**Table 2:** Performance of the AP4 relapse test in validation cohorts

Cohort	% AP4-	5 year survival (95% CI)	10 year survival (95% CI)	Hazard ratio (95% CI)	Logrank *p*-value
OXFD+	38%	0.96 (0.90 – 1.00)	0.93 (0.86 – 1.00)	6.73 (2.02 – 22.4)	3 × 10^-4^
MZ+	47%	0.92 (0.86 – 0.98)	0.85 (0.75 – 0.95)	2.23 (1.02 – 4.87)	0.039
GUYT+	31%	0.96 (0.89 – 1.00)	0.88 (0.76 – 1.00)	3.09 (0.90 – 10.5)	0.058
GUYT2+	36%	No metastases in AP4-			

TOTAL	40%	0.95 (0.93 – 0.98)	0.89 (0.85 – 0.93)	3.76 (2.16 – 6.56)	5.14 × 10^-7^

The 5 and 10 year metastsis-free survival probabilities for the AP4- groups in the validation cohorts, the hazard ratio for AP4- to AP4+, and the logrank *p*-value.

**Figure 4:**
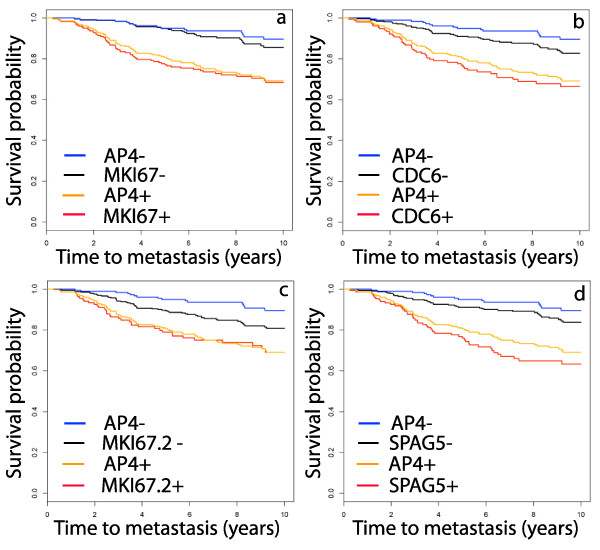
**Kaplan-Meier survival plots for the individual genes in AP4**. The partition defined by each of the AP4 genes individually is a significant predictor of metastasis, however each is less significant than AP4. In (a), *MKI67 *denotes the probe 212022_s_at and in (c), *MKI67.2 *denotes the probe 212020_s_at. The sample set is the same set used in Figure 3, namely, the union of the ER+ samples in OXFD, GUYT, MZ and GUYT2. The hazard ratios, with 95% confidence intervals, for these genes are: *MKI67*, 2.90 (1.84 – 4.57); *CDC6*, 2.47 (1.62 – 3.75); *MKI67.2*, 1.84 (1.20 – 2.82); *SPAG5*, 2.91 (1.92 – 4.43). These are significantly smaller than the hazard ratio of 3.76 for AP4.

The AP4 test improves on the prognostic power of each of the individual probes in the test. A preliminary step in calculating AP4 is a partition of the samples in a cohort into *CDC6 *+/-, *MKI67 *+/-, etc. The binary variables representing these partitions can be merged to represent partitions for each probe ranging over the full validation set. The Kaplan-Meier plots for each probe, juxtaposed with the AP4+/- plot, are found in Figure 4. While each probe yields a significant partition, none of the probes is as significant as AP4, as measured by the hazard ratio.

#### The classification of a sample as AP4- or AP4+ is stable across reference sets

In a generalization of the AP4 test beyond this study a new sample would be classified as AP4+ or AP4-using cutoffs defined with a reference set of samples. The stability of these cutoffs is tested with the following Monte Carlo cross-validation method. Given a microarray dataset for a cohort *S *and a subset of samples *S*_0 _balanced for relapse, thresholds are determined for the 4 probes in AP4 using the expression vectors restricted to *S*_0_. A sample not in *S*_0 _is classified as AP4+ or AP4- using the cutoffs defined in *S*_0 _and this status is compared to that calculated using all of *S*. We associate to *S*_0 _the fraction of correctly classified samples. The accuracy of the cutoff estimation is the mean value over a large number of subsets like *S*_0_. In this study we use 1, 000 subsets.

The accuracy of the reference set prediction of AP4 status, calculated across all cohorts, is 0.97 (95% CI 0.88 – 1.00). This high degree of stability is likely due to the intrinsic nature of the cutoffs.

#### AP4 improves on the prognostic power of clinical variables

A biomarker for relapse is only useful if it improves on the prognostic power of the standard clinical variables, such as tumor grade, size and lymph node status. We show that AP4 is significant in multivariate analysis and in stratified analysis on clinically defined subtypes. This study is performed on the 738 samples in the study for which data is available on distant metastasis, tumor grade, size and lymph node status. Tumor size is represented here by a binary variable that is 0 for tumors < 2 cm in diameter and 1 for tumors ≥ 2 cm.

The AP4 test improves on the clinical variables in a multivariate Cox proportional hazard model. The *p*-values for the clinical variables in univariate models are: grade, *p *= 5.6 × 10^-5^; node status, *p *= 0.02; size, *p *= 4.6 × 10^-7^. The *p*-value for grade, node status and size together is 9.0 × 10^-8^, while adding AP4 to these 3 gives *p *= 2.4 × 10^-15^. Comparing log-likelihoods, the level of significance of AP4 over grade + node status + size is 5.8 × 10^-11^. Note that the distribution of lymph node status in the full dataset is distorted by the fact that some cohorts contain only node negative samples (see Table 1).

**Figure 5:**
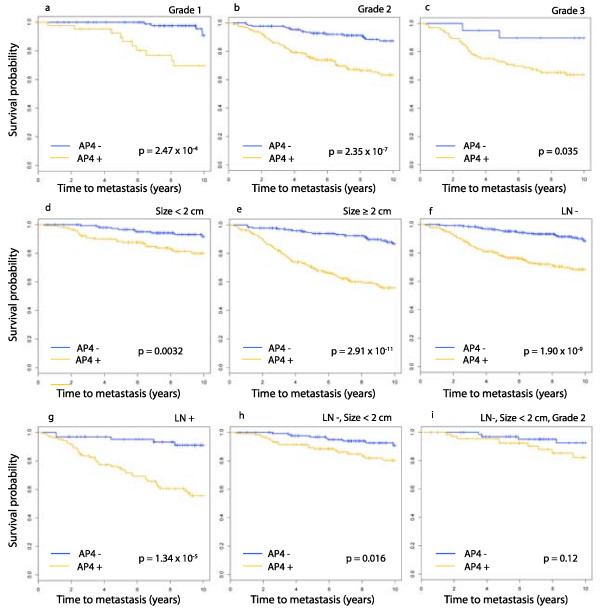
**Kaplan-Meier survival plot for AP4 in clinical subtypes**. The domain for this analysis is the set of 738 samples for which data exists on distant metastasis, grade, tumor size and node status. Each plot is for AP4 in the subtype indicated above the panel.

AP4 is found to be statistically significant on each of the subtypes defined individually by grade, size and lymph node status. The Kaplan-Meier plots are found in Figure 5(a)–(g). Keeping in mind that the goal of this project is to identify patients who may not benefit from chemotherapy, good prognosis groups are formed by combining clinical subtypes. AP4 is statistically significant on the set of lymph node negative tumors that are < 2 cm in diameter (Figure 5(h)). On the grade 2 tumors in this latter subgroup the *p*-value for AP4 is not below the significance threshold, however the Kaplan-Meier plot (Figure 5(i)) does show a pronounced divergence in expected survival for AP4- and AP4+. Most of the sets formed by intersecting three clinical subgroups are too small for meaningful analysis. It is worth noting that the 10-year expected survival probability in AP4- is nearly constant across all of these clinical subgroups, even poor prognosis groups such as grade 3 or LN+.

These clinical variables do not improve on the prognostic power of AP4-. In fact, tumor grade is not a significant predictor of metastasis on AP4- or on AP4+; i.e., the ability of tumor grade to predict metastasis is completely captured by AP4+/-. Lymph node status has the same relationship. Tumor size is not a significant predictor of metastasis on AP4-, but is significant on AP4+ (*p*-value = 7.56 × 10^-6^.)

## Discussion

In this study, a discrete model for metastasis in ER+ breast cancer was derived using genes that define intrinsic subtypes. The good prognosis group in the model, AP4-, has a high 10-year metastasis-free survival probability independent of clinical traits such as tumor grade, size and lymph node status. There is a high degree of stability in the boundary between AP4- and AP4+, likely due to the intrinsic nature of the cutoffs.

**Figure 6:**
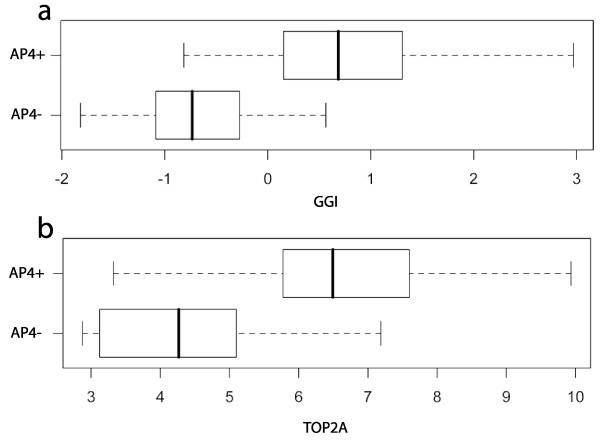
**Comparison of AP4 and indicators of chemo-sensitivity**. The distribution of GGI (a) and *TOP2A *(b) across the AP4+/- groups suggests that chemo-sensitive tumors are AP4+. The plot for GGI was calculated in the ER+ samples in TRANSBIG, while the plot for *TOP2A *is from MZ. The GGI values were scaled to a mean of 0 and standard deviation of 1.

While a prospective trial is needed to verify that AP4- patients do not significantly benefit from chemotherapy, this hypothesis is supported by the data in Table 2 and studies of chemo-sensitivity; i.e., the likelihood that a tumor will respond completely to chemotherapy. The NSABP B-20 trial [1] reports that ER+ node-negative patients receiving cyclophosphamide, methotrexate, fluorouracil and tamoxifen have a 12-year overall survival probability of 0.87. This probability is comparable to the AP4- 10-year metastasis-free survival probability of 0.89 (95% CI 0.85 – 0.93). However, it is important to know that the AP4- tumors that eventually metastasize are not those that will benefit the most from chemotherapy. The genomic grade index (GGI) [20], a test for recurrence in ER+ breast cancer that is highly enriched with cell cycle progression genes, is also correlated with chemo-sensitivity [21]. As Figure 6(a) shows, only a few AP4- samples have GGI values above the mean of 0. Moreover, *TOP2A*, a target for anthracyclines [22], is expressed at a low level throughout AP4- tumors, Figure 6(b). Thus, those tumors that respond most favorably to chemotherapy because they have high proliferation rates are likely to be AP4+. This makes it less likely that chemotherapy will benefit the AP4- patients.

The methodology leading to the AP4 test actually identifies a family of models with comparable hazard ratios in the samples used here. These alternatives use 4 to 7 probes, chosen from those defining AP4 and probes representing *CDT1 *(chromatin licensing and DNA replication factor 1), *PLK1 *(polo-like kinase 1), *CDC45L *(CDC45 cell division cycle 45-like (S. cerevisiae)), and *SNRPA1 *(small nuclear ribonucleoprotein polypeptide A'). Most of these genes are directly involved in mitosis, consistent with the central role of cell cycle progression in ER+ breast cancer relapse [20,23]. Reports of poor prognosis in carcinomas with elevated expression of these genes are widespread [22,24-27]. While *MKI67 *and *CDC6 *have been widely studied, the other gene in the AP4 test, *SPAG5 *is less well-known. Also known as *Astrin*, *SPAG5 *codes a protein involved in mitotic spindle assembly. Silencing of *SPAG5 *induces p53-mediated apoptosis and sensitizes cells to paclitaxel treatment in HeLa cells [25]. In [28] it is shown that SPAG5 interacts with AURKA (STK15). Both *MKI67 *and *AURKA *are found in the Oncotype DX panel.

Using the results in this study, the AP4 test could be implemented with a reference set of microarrays. A patient would be tested by hybridizing mRNA from the tumor to a microarray, applying gcrma to this microarray and the reference set together, and determining the sample's AP4 status using cutoffs determined with the reference samples. However, full genome microarrays are comparatively expensive and generate a huge amount of information that is not used in determining AP4 status. The development of a clinically useful form of the AP4 test requires (1) selection of a method for measuring the mRNA concentration or protein levels of the associated genes, (2) analysis of the density distribution of these measures and selection of cutoffs using the mixture model method, and (3) determination of the long-term expected survival probability for the AP4- group calculated using the cutoffs from (2). While the most direct method would use RT-PCR or a custom microarray to measure the mRNA levels, it is likely that some test in the accelerated progression family can be implemented with IHC. This hypothesis is supported by other studies of the genes in the accelerated progression family. The Ki-67 proliferation index for a tissue sample is the percentage of cells that respond positively to the MIB-1 antibody using IHC, also called the labeling index for Ki-67 [22,29]. The density distribution of Ki-67 labeling indices in [[30], Figure 1] shows a pronounced tail to the right, similar to gene expression for *MKI67 *(see Figure 1(b)). Refer also to results on CDC6 [31], CDC45L [26], and PLK1 [27]. Such a test could use commonly available formalin-fixed, paraffin-embedded tissue samples. We anticipate validating this more clinically useful test in a retrospective study on an independent set of samples.

An additional concern in focusing solely on the few genes in this test is that the expression values in the microarray datasets may be distorted by nonspecific binding of other mRNA species to the Affymetrix probes. This problem would reveal itself through a density distribution under (2) that is very different from that found in the array data. Whether this distortion occurs at a significant level can only be determined through step (3) above.

Previous studies of several of the genes used in the accelerated progression family of models report that tumor tissues have two distinct expression patterns of these genes, supporting our multi-state methodology. Expression of *CDC6 *in non-small cell lung cancer cells, as assessed with RT-PCR, partitions cells into two groups; one with baseline expression, and a second group with highly elevated expression [[31], Figure 2]. Similar patterns were found for *CDT1 *[31]. This same paper reports that IHC with an antibody for CDC6 can also discriminate the low and high expression states for that gene. Using IHC, PLK1 was detected at a high level in invasive carcinomas of the breast and undetected in normal breast tissue [27].

**Figure 7:**
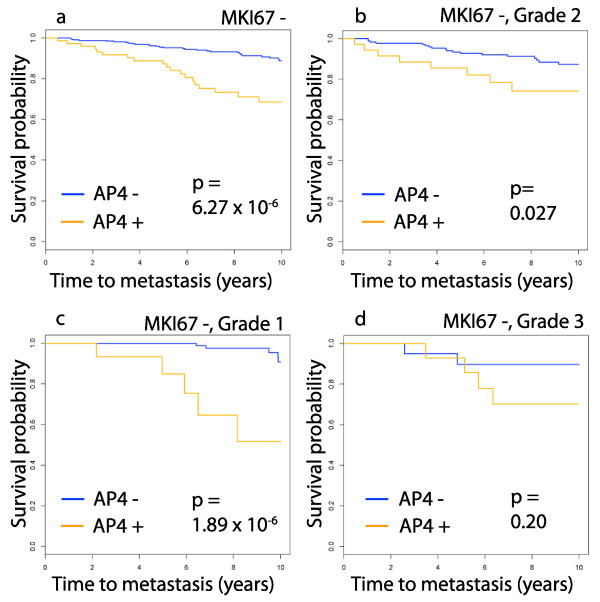
**Survival plots for AP4 in subtypes defined by proliferation markers**. Here, the *MKI67 *+/- partition is used as a surrogate for the Ki-67 proliferation index. Combining this with tumor grade gives subtypes defined by clinical proliferation markers. The domain for this analysis is the same set of 738 samples used in the study of clinical subtypes. The subtypes are indicated above the panels. AP4 improves on the prognostic power of these subtypes, except on the small group of grade 3 tumors that are *MKI67 *-. AP4 is vacuous on *MKI67 *+ since all such samples are AP4+.

Although the genes comprising the AP4 test are central to mitosis, the test apparently improves on the prognostic power of common proliferation markers such as the Ki-67 proliferation index and tumor grade. Indeed, the presence of *MKI67 *in a test that uses few genes, such as AP4, requires validating that the test has greater prognostic power than Ki-67. Here, AP4 is compared to a proliferation indicator using the *MKI67 *+/- partition (using 212022_s_at) as a surrogate for the Ki-67 proliferation index. On the good prognosis group *MKI67 *-, the *p*-value of AP4 is 6.27 × 10^-6 ^(Figure 7(a)). The Kaplan-Meier plots of AP4 on the grade 1, 2 and 3 tumors in *MKI67 *– (Figure 7(b)–(d)) show that AP4 is significant in all of these subtypes, except for the grade 3, *MKI67 *-, tumors. This latter group contains only 35 samples.

The Oncotype DX test utilizes genes involved in processes besides proliferation, however the dominance of proliferation genes in ER+ breast cancer progression is widely reported [20,23]. The 16 genes in the Oncotype DX panel are grouped as proliferation genes, tumor invasion genes, those related to *HER2 *and estrogen, and three others. Certainly, processes besides proliferation contribute to breast cancer metastasis, however this does not indicate that proliferation markers are improved by additional genes.

A comparison with the proliferation group in Oncotype DX suggests that AP4 contains significantly more prognostic power than that group. The contribution of the proliferation genes to the Oncotype DX recurrence score is modeled here by the mean of the expression values of the probes 202095_s_at (*BIRC5*), 212022_s_at (*MKI67*), 201710_at (*MYBL2*), 214710_s_at (*CCNB1*), 204092_s_at (*AURKA*), which we call the *proliferation score *(see [[4], Figure 1]). To compare this surrogate proliferation score with AP4, we find the high-low partition of the score that best approximates the AP4+/- partition. These binary proliferation scores, for each of the four validation cohorts, are concatenated to form a binary variable, ranging over the entire validation set. Comparing CPH models using the binary proliferation score alone, and this variable together with AP4, shows that AP4 significantly improves on the binary proliferation score with a *p*-value of 2.37 × 10^-5^. Thus, even though the genes defining AP4 are central to proliferation they seem to contain significantly more information about metastasis than the proliferation group in Oncotype DX.

**Figure 8:**
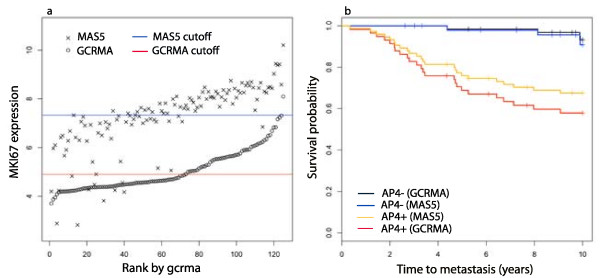
**Comparison of models defined with gcrma and MAS5**. Microarray data from a set of 125 samples in TRANSBIG+ was normalized with both gcrma and MAS5 on a log2 scale. In (a) the samples were rank ordered by gcrma expression values of *MKI67 *(212022_s_at) and these values are plotted. The log2 MAS5 expression values for these samples are also plotted. The red line indicates the +/- cutoff for the gcrma values and the blue line the cutoff for MAS5 values, as calculated by the multi-state methods. The calculation of AP4 is completed on both sets of expression values to produce gcrma and MAS5 versions of the test. (b) The Kaplan-Meier plots for the two versions of AP4 are given. The gcrma version of the test has a hazard ratio of 8.83 (95% CI 3.06 – 25.2), while the MAS5 version has a hazard ratio of 4.67 (95% CI 1.62 – 13.5).

Using gcrma rather than the more common MAS5 to calculate expression values gives a more powerful AP4 test. The genes in the accelerated progression family are unexpressed or expressed at a low baseline level in normal breast tissue, so accurate and precise measurements of low mRNA concentrations are important. It was shown in [18] that gcrma is more precise than MAS5 in measuring mRNA at low concentration levels. To explore this issue we normalize 125 samples in TRANSBIG+ with MAS5 and apply the mixture model methods to calculate thresholds for the AP4 genes and a MAS5 version of the AP4+/- partition. In Figure 8(a) expression values for *MKI67 *(212022_s_at) computed with gcrma are plotted in increasing order, along with the values computed with MAS5, on a log2 scale, and the cutoffs for *MKI67 *+/- under both methods. As the figure shows, MAS5 measures for samples that have low gcrma values, have significantly higher variance. Many of these samples are classified as *MKI67 *+ under MAS5. Similar behavior is found in the other AP4 probes. Under MAS5, 50 samples are AP4- and all but one of these are AP4- under gcrma. However, gcrma classifies an additional 18 samples as AP4-. As Figure 8(b) shows, the long-term expected survival of the two AP4- groups are comparable. Thus, gcrma is able to identify significantly more good prognosis patients than MAS5.

Studies of Oncotype DX and Mammaprint report that the good prognosis groups they predict consist of 40% to 50% of the study population [7,32]. The AP4- group in the validation set consisting of the ER+ samples in OXFD, GUYT, GUYT2 and MZ contains 40% of the samples. However, Mammaprint and Oncotype DX are only administered to node negative patients. Of the node negative samples in the validation set, 43% are AP4-. In all node negative and ER+ samples in this study, 49% are AP4-.

The properties of the expressed sequence tags (EST) targeted by the two probes for *MKI67 *deserve further study. The probe most significantly connected to relapse is 212020_s_at, which hybridizes the EST GenBank: AU152107. This is transcribed from a continuous block of DNA in Exon 1 of *MKI67 *(see UCSC Genome Browser http://genome.ucsc.edu). The second probe, 212022_s at targets GenBank: BF001806, transcribed from 3 blocks of DNA in Exon1, Exon 2 and Exon 3. It is unclear whether the corresponding mRNA code for different isoforms of the MKI67 protein. The fact that they are both significantly correlated to metastasis suggest that they both code functional proteins. It is known that *MKI67 *is found in multiple isoforms that effect cellular processes differently [33].

## Conclusion

The accelerated progression relapse test is defined using few genes and a minimal amount of statistical modeling. The thresholds between the low and high components of the genes define stable subtypes correlated with metastasis across independent cohorts. This test identifies a group of ER+ tumors that are unlikely to respond to adjuvant chemotherapy.

## Abbreviations

ER: estrogen receptor; MKI67: antigen identified by monoclonal antibody Ki-67; CDC6: cell division cycle 6 homolog (S. cerevisiae); SPAG5: sperm associated antigen 5; ERBB2: v-erb-b2 erythroblastic leukemia viral oncogene homolog 2, neuro/glioblastoma derived oncogene homolog (avian); CPH: Cox proportional hazard model; IHC: immunohistochemistry; ESR1: estrogen receptor 1; GGI: genomic grade index; CDT1: chromatin licensing and DNA replication factor 1; PLK1: polo-like kinase 1; CDC45L: CDC45 cell division cycle 45-like (S. cerevisiae); SNRPA1: small nuclear ribonucleoprotein polypeptide A'; AURKA: aurora kinase A; RT-PCR: reverse transcription-polymerase chain reaction; BIRC5: baculoviral IAP repeat-containing 5 (survivin); MYBL2: v-myb myeloblastosis viral oncogene homolog (avian)-like 2; CCNB1: cyclin B1

## Competing interests

The University of Notre Dame has filed a provisional patent on behalf of Steven Buechler for the results in this paper.

## Authors' contributions

All research and manuscript preparation was done by Steven Buechler. All authors read and approved the final manuscript.

## Pre-publication history

The pre-publication history for this paper can be accessed here:

http://www.biomedcentral.com/1471-2407/9/243/prepub
